# Response evaluation of two commercial thermoluminescence dosimeters (TLDs) against different parameters

**DOI:** 10.1259/bjro.20220035

**Published:** 2023-04-19

**Authors:** Sitah Fahad Alanazi, Haya Alarifi, Abdullah Alshehri, Mansour Almurayshid

**Affiliations:** 1 Department of Physics, College of Science, Imam Mohammad ibn Saud Islamic University (IMSIU), Riyadh, Saudi Arabia; 2 Nuclear Technologies Institute, King Abdulaziz City for Science and Technology (KACST), Riyadh, Saudi Arabia

## Abstract

**Objectives:**

It is essential to study the dosimetric performance and reliability of personal dosimeters. This study examines and compares the responses of two commercial thermoluminescence dosimeters (TLDs), the TLD-100 and the MTS-N.

**Methods:**

We compared the two TLDs to various parameters such as energy dependence, linearity, homogeneity, reproducibility, light sensitivity (zero point), angular dependence, and temperature effects using the IEC 61066 standard.

**Results:**

The results acquired showed that both TLD materials show linear behavior as indicated by the quality of the ﬁt. In addition, the angular dependence results for both detectors show that all dose responses are within the range of acceptable values. However, the TLD-100 outperformed the MTS-N in terms of light sensitivity reproducibility for all detectors together, while the MTS-N outperforms the TLD-100 for each detector independently and that showed TLD-100 has more stability than MTS-N. The MTS-N shows better batch homogeneity (10.84%) than TLD-100 (13.65%). The effect of temperature in signal loss was clearer at higher temperature 65°C and it was however below ±30%.

**Conclusions:**

The overall results for dosimetric properties determined in terms of dose equivalents for all combinations of detectors are satisfactory. The MTS-N cards have better results in the energy dependence, angular dependency, batch homogeneity and less signal fading, whereas the TLD-100 cards are less sensitive to light and more reproducible.

**Advances in knowledge:**

Although previous studies showed several types of comparisons between TLDs, they have used limited parameters and different data analysis. This study has dealt with more comprehensive characterization methods and examinations combining TLD-100 and MTS-N cards.

## Introduction

Employees dealing with ionizing radiation in the research, industrial and medical field could be exposed to low doses. The usage of radiation in experiments, industrial radiography, and medical examinations and treatments is continuously increasing. There is a linear relationship between a person’s exposure to radiation and the development of cancer. As a result, accurate measurements of the accumulated doses absorbed by employees are required.^1^ International Commission of Radiological Protection (ICRP) prescribed limits per annum. For example, a member of the public should not receive more than that 1 mSv per annum from background for the whole body, 5 mSv to eyes, and 20 mSv to hands. Whereas radiation workers should not receive more than that 20 mSv per annum, 150 mSv to eye and 500 mSv to hands.

Thermoluminescent dosemeter (TLD) is a personal dosimetry used to measure exposure to radiation doses. TLDs have a number of advantages that make them easy to use and reproduce in clinical and research settings, including their small size, reusability, and the need for few correction factors, among others.^2–6^ The TLD consists of ships of a certain type of material such as lithium fluoride or calcium fluoride worn when dealing with radiation environment. It is used to calculate the accumulative amount of exposure to radiation over a period of time for radiation protection purposes.^1^

The thermoluminescence (TL) physical principle may be defined as a two-way process. The first stage involves the absorption of energy from ultraviolet (UV) or ionizing radiation, which transforms the system from equilibrium to metastable. The second stage involves bringing the system back to equilibrium by releasing energy, such as light, with the assistance of thermal stimulation (TLD reader). Following the absorption of energy from radiation, TL is the thermally stimulated emission of light.^7,8^ Because their property is tissue equivalent, (LiF: Mg, Ti), (LiF: Mg, Cu, P), and (Li2 B4 O7 : Mn) are the most commonly utilized TLDs in radiation-based applications.^9–11^

TLDs can be used to determine a precise personal equivalent dose, especially if the detector is comparable to a tissue-equivalent material (*Z _eff, tissue_* = 7.4). Due to its close similarity to human tissues, the phosphor lithium fluoride (LiF) doped with Mg and Ti (LiF: Mg, Ti) was the most investigated and utilized TLD material in for health and medical physics dosimetry applications (*Z _eff, LiF_* = 8.2).^1^

Many companies manufacture TLDs and we have chosen to compare two commercial TLDs for this study: MTS-N made by Radcard (Fabryczna, Kraków, Poland) and TLD-100 made by Thermo Scientific^™^ (Waltham, Massachusetts, USA).^12,13^ Although both TLDs made from the same materials of LiF: Mg, Ti, the productions of these two TLD detectors are entirely different technologies. The technology used to manufacture TLD-100 is through extruding single crystals through a rectangular die at 700°C. The MTS-N is produced by Institute of Nuclear Physics (INP) via sintering a polycrystalline LiF powder activated with Mg and Ti at the stage of its chemical synthesis. TLD-100 is considered most models of TLD employed in experiments and radiation protection purposes. Most studies relate the response or compare the performance of any detector under investigation to TLD-100. However, the only drawback for TLD-100 at this stage is that it is relatively more expensive than MTS-N [^13^].

The aim of this study is the comprehensive comparison of the responses of two TLDs by evaluating the TLD signals against many parameters such as dose linearity, reproducibility, effect of light exposure on the and stability to insure that the accurate measurements and the results would be compared to the tolerances and acceptance criteria provided in the international electro technical commission (IEC) 61066.^14^ In addition, the effect of ambient temperature in signal fading would be studied in lab-scale measurements. The outcome of the study would affect our decision of our next purchase TLDs to be distributed for workers and clients.

## Methods and materials

### Experimental setup

Experiments were carried out in the radiation calibration laboratory and the data was read in the personal dosimetry measurement laboratory, where the Harshaw TLD Model 6600 Plus Reader made by Thermo Scientific^™^ (Waltham, Massachusetts, USA) was used to read all dosimeters using the manufacture recommended parameters in Table 1. All tlds used were calibrated in the study, to ensure that all the dosimeters in the system will provide virtually the same response to a given radiation dose by determining the acceptable element correction coefficient (ECC) range for the dosimeters.

**Table 1.** Time-temperature profile (TTP) used for TLD-100 and MTS-N

Parameters	TLD-100	MTS-N
**Preheat Temperature (°C**)	50	170
**Preheat Time (s**)	0	5
**Acquire Temperature (°C**)	300	300
**Acquire Time (s**)	13.33	13.33
**Annealing Temperature (°C**)	300	300
**Annealing Time (s**)	0	5

**Figure 1.**
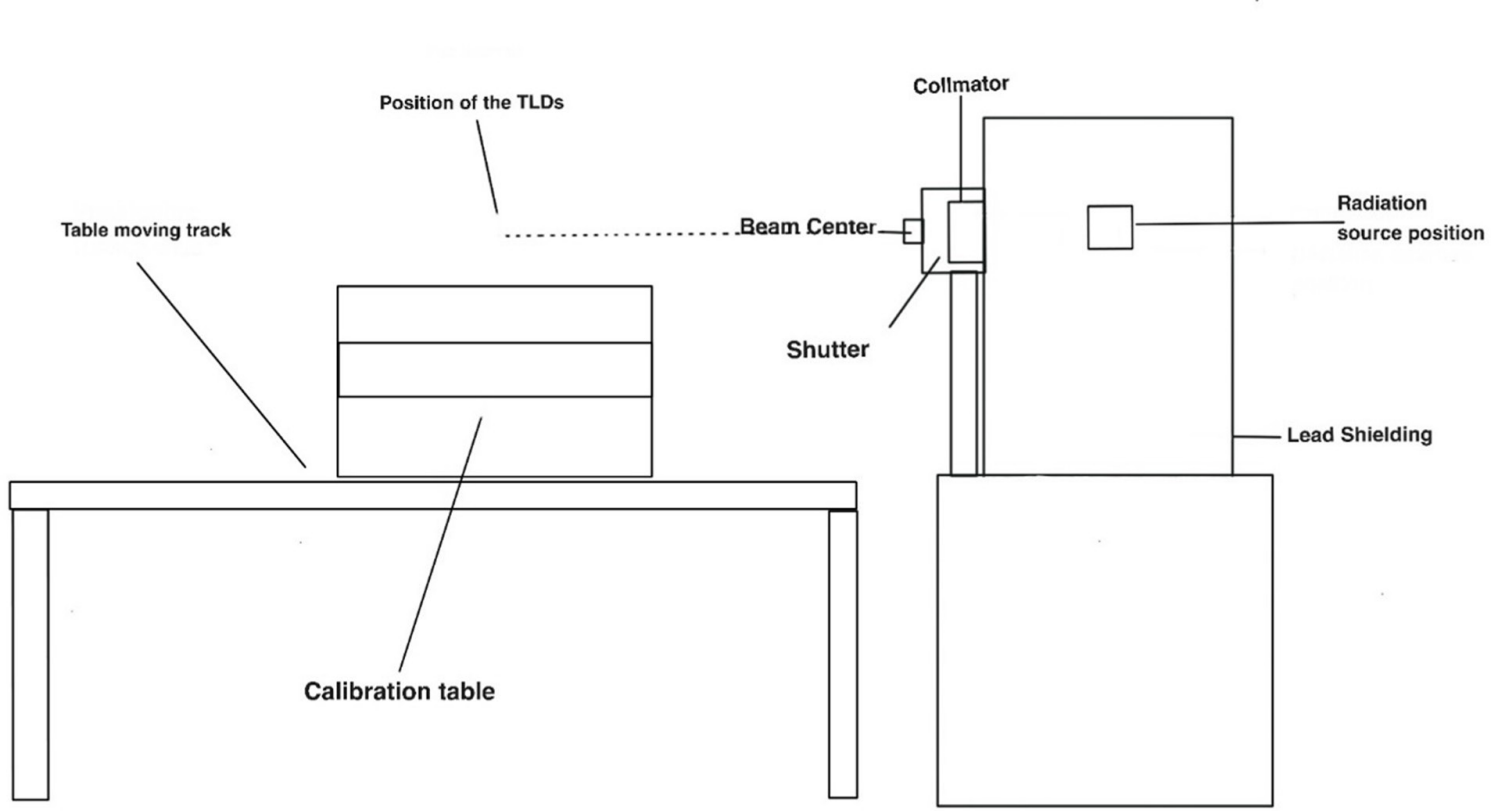
Schematic sketch of the irradiation systems used in four experiment where the beam (γ or X-rays) is formed and collimated five to irradiate the TLDs at 100 cm away from the radiation source unless it is six stated.

Figure 1 shows a diagram of the overview of the prototype used to irradiate the TLDs. When stated, we used γ rays to irradiate the TLDs in this work by using Cs-137 or a 320 *kV* x-rays machine manufactured by Gulmay Limited (Byfleet, UK) that produces x-rays. Both systems are connected and controlled remotely in a separate control rooms.

### Thermoluminescent dosemeter (TLD)

Lithium ﬂuoride Thermoluminescent phosphor is typically considered as a superior material for a number of dosimetric applications, especially when used as solid pellets. It has a wide range of applications because to its high sensitivity, low background, environmental resistance, and tissue equivalency. We used two types of TLDs in this study: the Thermo Scientific^™^ TLD-100 (USA) and the RADCARD MTS-N (Poland).

#### Thermo scientific™ TLD-100

**Figure 2.**
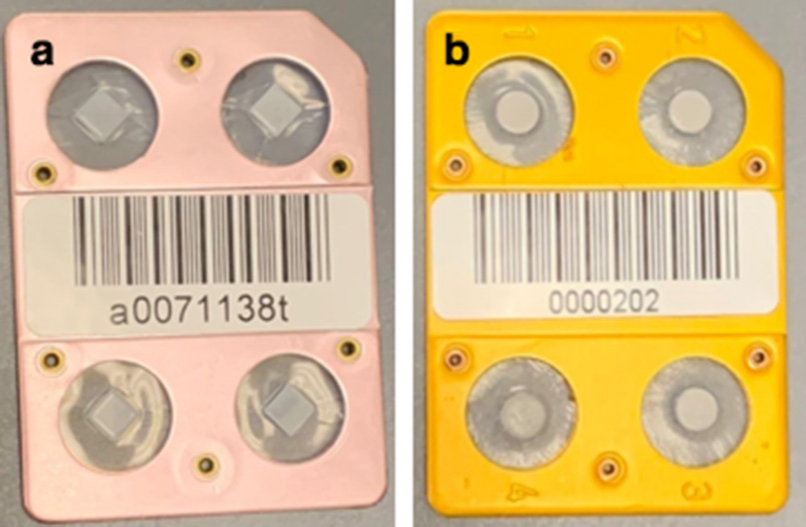
Demonstration of the TLDs used in this study (**A**) TLD-100 dosemeter (**B**) MTS-N dosemeter

LiF: Mg, Ti are all alkali halides that are commonly used as personal dosimeters. Powders, cubical or cylindrical chips, rods, and other TLD dosimeters are commonly used. TLD100 chips are LiF crystals that have been doped with titanium and magnesium in order to increase the number of traps and luminescence centers. In medical and environmental dosimetry, TLD100 chips are widely used. For the experiments, cube-shaped TLD chips with dimensions of 3.1× 3.1 mm × 1 mm were used as shown in Figure 2-a.^15^ It has an effective atomic number of 8.2 and density of 2.64 g cm^−3^.

### Radcard mts-n

The RADCARD MTS-N (LiF: Mg, Ti) is produced as solid pellets with a diameter of 4.5 mm and a thickness of 0.2–1.5 mm (standard 0.9 mm) under the code name MTS (Mg, Ti, sintered), with an additional symbol reﬂecting the lithium isotope content: MTS-N (natural abundance), MTS-6 (6Li-enriched), MTS-7 (7Li-enriched) as shown in Figure 2-b.^16^ It has an effective atomic number of 8.2 and density of 2.50 g cm^−3^.

### 3.experimental tests

Thermo Scientific^™^ TLD-100 and RADCARD MTS-N cards were compared using some selected tests from the IEC 61066 standard. The comparative tests included the following: energy response, linearity, batch homogeneity, reproducibility, light sensitivity (zero point), angular dependence and effect of temperature in signal fading.

#### Energy response

For the determination and comparison of the energy response between TLD-100 and MTS-N, both were irradiated simultaneously in order to investigate their energy response. Three groups of each type were prepared, each containing four cards of TLDs. The three groups were irradiated at an effective photon energy of 165 keV using the X-ray irradiation system with different doses of 2, 5, and 10 mSv, and as with the previous one, the other three groups were irradiated with a Cs-137 source (≈ 662 keV) with similar doses as the previous. For x-rays, the applied voltage and the current values were set to 200 kV and 15 mA, respectively. The additional filtration was 1 mm Pb, 3 mm Sn, 3 mm Cu and 4 mm Al selected in accordance to the narrow beam quality descripted in ISO 4037 used to conduct radiation calibrations.^17^ The TLD measurement was carried out using a Harshaw 6600 Plus TLD reader. The measured responses for the energy response test must not deviate more than 30% from the true value of dose, as required by the IEC 61066 according to the following relation shown below.^11^

(1) $$0.7\leq \frac{\overset{-}{E_{i}+I_{i}}}{C_{i}}\leq 1.3$$

where $\overset{-}{E_{i}}$ is the mean of the response, C_i_ is the true dose, and I_i_ denotes the confidence interval, which is determined as I_i_ = $\frac{t_{nS_{i}}}{\sqrt{n_{i}}}$ . Where Si is the standard deviation for the *i-th* group of measurements, t_n_ is a value for confidence intervals for n_i_ measurements.

### Linearity of the measured signal versus the delivered radiation

In the linearity test, three groups of each type were prepared, each containing six dosimeters; half of them were TLD-100 and the other half were MTS-N. The three groups were given speciﬁc doses of 1, 10, 50, and 100 mSv of the Cs-137 source. The measured responses for the linearity test must not deviate more than 10% from the true value of dose, as required by the IEC 61066 according to the following relation shown below.^11^

(2) $$0.9\leq \frac{\overset{-}{E_{i}+I_{i}}}{C_{i}}\leq 1.1$$

### Batch homogeneity

For measuring the batch homogeneity six groups were prepared, each containing 30 dosimeters. To determine the individual sensitivity factor for the homogeneity test, three groups for each TLD type. TLD-100 was irradiated with doses of 1, 2, and 3 mSv from a Cs-137 source, similar doses were used to irradiate the other type. According to the following relation shown below, we calculate batch homogeneity

(3) $$\frac{E_{max}-E_{min}}{E_{min}}\leq 0.3$$

where E_max_ is the maximum of the response, and E_min_ is the minimum of the response.

### Reproducibility

Detector reproducibility refers to a detector’s ability to maintain stable readings after successive use.^18^ The criteria state that the coefficient of variation of the evaluated value shall not exceed 7.5% for each dosemeter separately.^11^ A group of three dosimeters was irradiated to 10 mSv of Cs-137 and read ten times, group for TLD-100 and the other for MTS-N. Using the following relationship, we can calculate the reproducibility coefficient of variation:

(4) $$\frac{S_{\overset{-}{E_{i}}+}+I_{i}}{\overset{-}{E_{i}}}\leq 0.075$$

where $\overset{-}{E_{i}}$ is the mean of the response, *I_i_* is the conﬁdence interval, and $S_{\overset{-}{E_{i}}}$ is the standard deviation.

### Light sensitivity (zero point)

Four groups of six dosimeters, two groups for TLD100 and two for MTS-N were irradiated with a 10 mSv dose. Two groups, one is TLD-100 and the other is MTS-N, were put under fluorescent lighting for a period of 24 h (h) of a daylight fluorescent lamp with intensity of around 5 W m^−2^, and the other two groups were set in dark conditions for a period of 24 h, and after that, all of them were readout. According to the following relation shown below, the zero point must not change by more than 0.1 mSv after 24 h.^14^

(5) $$\left[\bar{E_{1}}-\bar{E_{2}}\;\right]\pm I<H\left(0.1mSv\right)$$

where $\overset{-}{E_{1}}$ represents the exposed group, $\overset{-}{E_{2}}$ represents the store in the dark group, I represents the confidence interval, and *H* = 0.1 mSv.

### Angular dependence

To describe dosemeter behavior in real world conditions, angular dependence laboratory tests are required. The dosimeters were tested by varying the angle of incidence in relation to the normal, from 60°, 40°, 20°, 0°, −20°, −40°, −60° for the Cs-137 radiation, two groups, one for TLD-100 and the other for MTS-N. Each group has 21 dosimeters, three for each angle, and they are irradiated with a dose of 10 mSv for each group of three at a different angle. The mean response at angles of incidence 60°, 40°, 20°, −20°, −40°, −60° shall not differ by more than 15% from the corresponding angle of incidence of 0°.^14^

### Effect of temperature in signal loss

Three temperatures were selected to account for high temperature environments that a TLD may be exposed to. The temperatures chosen for this study were 25°C (control), 45°C, and 65°C. These temperatures, respectively, represent a hot day in Saudi Arabia. TLDs dosimeters were exposed to 1, 2, and 3 mSv doses irradiated with Cs-137 and after that put in the oven for 24 h after that readout. Each dose has three groups for different temperatures: 25°C, 45°C, and 65°C, so eventually we have nine groups to study the effect of temperature on the TLD-100 and the MTS-N. For the TLD-100 groups, each one was given a dose of 1, 2, and 3 mSv, similarly for the MTS-N groups as the previous one. Since each TLD has a slightly different radiation sensitivity, each TLD was compared to the TLDs to the room temperature, which is considered the control group with the same dosemeter in different temperature groups.^19,20^

## Results and discussion

### Energy response

The mean relative response of the observed TLD doses for TLD-100 and MTS-N when irradiated by Cs-137 and X-ray, respectively, is shown in Table 2. The TLD-100 and MTS-N mean relative response values for the groups that were irradiated with Cs-137 and x-rays with doses of 2, 5, and 10 mSv are shown in Table 2. For Cs-137, TLD-100 has a maximum diversion of true dose of about 1.32 at 10 mSv dose, whereas MTS-N has a maximum value of 1.06 at 2 mSv. For X-rays, all the responses were below 30% diversion where the maximum value of diversion from the true doses were 1.29 for TLD-100 at 5 mSv dose, and 1.17 for MTS-N at 10 mSv dose. According to the results, TLD-100 has a higher relative response than MTS-N when using 10 mSv of Cs-137 by about 25% and when using 5 mSv of X-rays by 10%. The IEC 61066 states that the coefficient of variation must not deviate from the conventional true value by more than 0.3.^8^ Therefore, the relative response of the TLD detector adequately satisfied the IEC 61066. The average difference in the energy dependency for the TLD-100 is approximately 20% and when using MTS-N, it is 9%. The MTS-N shows better energy dependency by 11% less than the TLD-100.

**Table 2.** Relative responses of TLD-100 and MTS-N irradiated by Cs-137 radiation and X-rays

True Dose (mSv)	662 keV Cs-137 irradiation		165 keV X-ray irradiation	
	Relative Response		Relative Response	
	TLD-100	MTS-N	TLD-100	MTS-N
2	1.19	1.06	1.20	1.13
5	0.96	1.01	1.29	1.16
10	1.32	1.01	1.18	1.17

### Linearity

**Figure 3.**
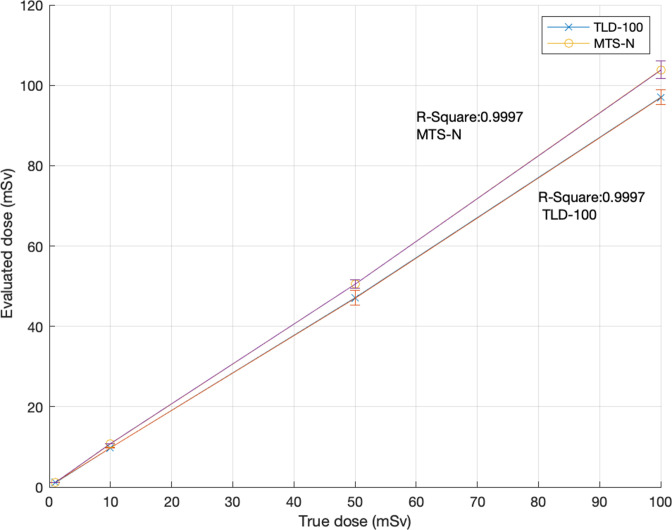
Linearity of the measured TLD signals against the delivered doses

The measured responses for the linearity test must not differ more than 10% from the true value of dose over the 1to 100 mSv range, as stated by the IEC 61066. Table 3 shows that the TLDs dosimeters match the IEC 61066 for the relative response in all doses except at 1 mSv, where it is more than 1.10 by 14% for MTS-N, when it should be between 0.9 and 1.10 according to the IEC 61066 criterion. It’s important to note that the high resultant readings, particularly at smaller doses, may be due to source uncertainty. As demonstrated by the quality of the fit, the results corresponding to linearity measurements in Cs-137 radiation beams for TLD-100 show linear behavior. MTS-N’s results are better because the slope is 1.033, which is closer to unity by 3.3 %, whereas TLD-100’s slope is 0.9658, which is 3.4% away from unity. The TLD-100’s y-intercept is 0.1057, while the MTS-is N’s 0.01503, indicating that the MTS-N has a better intercept than the TLD-100, which is over double the MTS-N’s value (Figure 3).

**Table 3.** The linearity relative response of the TLD-100 and MTS-N

True dose (mSv) Relative response		
TLD-10	1 10 50 100	1.10 0.99 0.97 1.02
MTS-N	1 10 50 100	1.14 1.09 1.02 1.09

### Batch homogeneity

The readings demonstrated a variation coefficient for TLD-100 and MTS-N, respectively, as shown in Table 4. For all dosages of 1, 2, and 3 mSv, all groups had values less than the maximum acceptable value of 30% stated by the IEC 61066.^8^ It’s worth noting that the high resultant values, particularly at lower doses, may be related to source uncertainty. The MTS-N shows better batch homogeneity by 10.84% less than TLD-100, which is 13.65%.

**Table 4.** Batch homogeneity of the personal dosimetry system of TLD- 100 and MTS-N

True Dose	1 mSv	2 mSv	3 mSv
Average Batch Homogeneity(TLD-100)	14.11%	12.27%	14.57%
Average Batch Homogeneity(MTS-N)	10.66%	12.06%	9.81%

### Reproducibility

**Figure 4.**
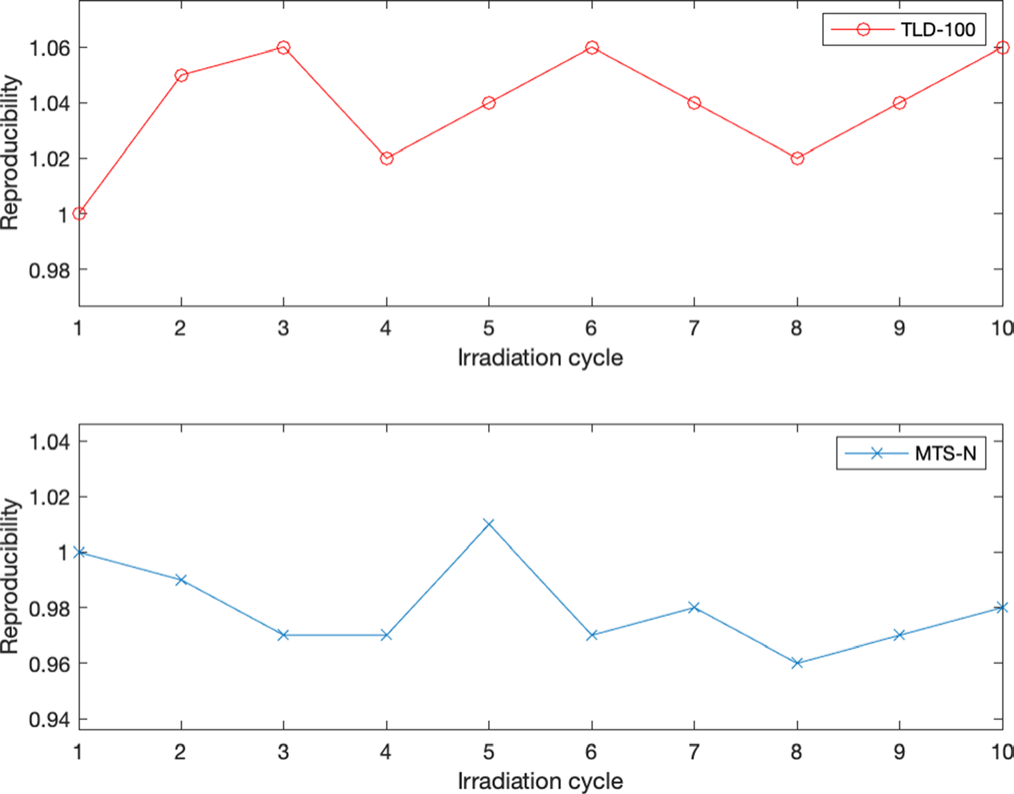
Reproducibility normalized to the first cycle following 10 irradiation cycles for the two detectors.

The coefficients of variation of the measured TL signals for each dosemeter separately and for all three detectors collectively for TLD-100 and MTS-N are shown in Tables 5 and 6. Using equation 4, the coefficients of variation for the TLD-100 detector did not exceed 0.033 separately, and the total coefficients of variation for all three detectors did not exceed 0.027. The coefficients of variation for the MTS-N, on the other hand, did not surpass 0.054 for each detector separately, and 0.024 for all three detectors collectively. According to the IEC 61066, the coefficient of variation must not exceed 0.075. TLD-100 provides higher stability than MTS-N for each detector separately, according to the data.^14^ Figure 4 presents the reproducibility of each TLD type, following the 10-irradiation cycles where the results were normalized to the first cycle.

**Table 5.** Reproducibility of TLD-100 card

Each dosemeter separately	
Dosemeter ID	Coefﬁcient of variation
7,0600	0.033
7,1147	0.031
7,0603	0.031
All dosemeter collectively Coefficient of variation	0.027

**Table 6.** Reproducibility of MTS-N cards

Each dosemeter separately	
Dosemeter ID	Coefﬁcient of variation
207	0.054
253	0.024
244	0.021
All dosemeter collectively Coefficient of variation	0.024

### Light sensitivity (zero point)

The light sensitivity of the dosemeter is determined using equation 5, which includes the confidence interval, which is dependent on the sample size and variations. A 10 mSv dose is used to irradiate four groups of TLDs, two for TLD-100 and the other two for MTS-N. For a 24 h period, Group one was exposed to fluorescent lights, whereas Group two was kept in the dark. We found that TLD-100 has −0.47 and −0.78 and meets the IEC 61066 criterion of less than 0.1 mSv after 24 h, whereas MTS-N has 0.25 and −0.14 slightly above that the IEC 61066 criterion of more than 0.1 mSv, which is consistent with the reproducibility results in which MTS-N TLD has more variations than TLD-100 (Table 7). Velbeck et al. (1999) showed similar behavior of the calculated results (positive and negative values).^21^

**Table 7.** Light sensitivity (zero point)

Material		
TLD-100	−0.47 *mSv*	−0.78 *mSv*
MTS-N	0.25 *mSv*	−0.14 *mSv*

### Angular dependence

**Figure 5.**
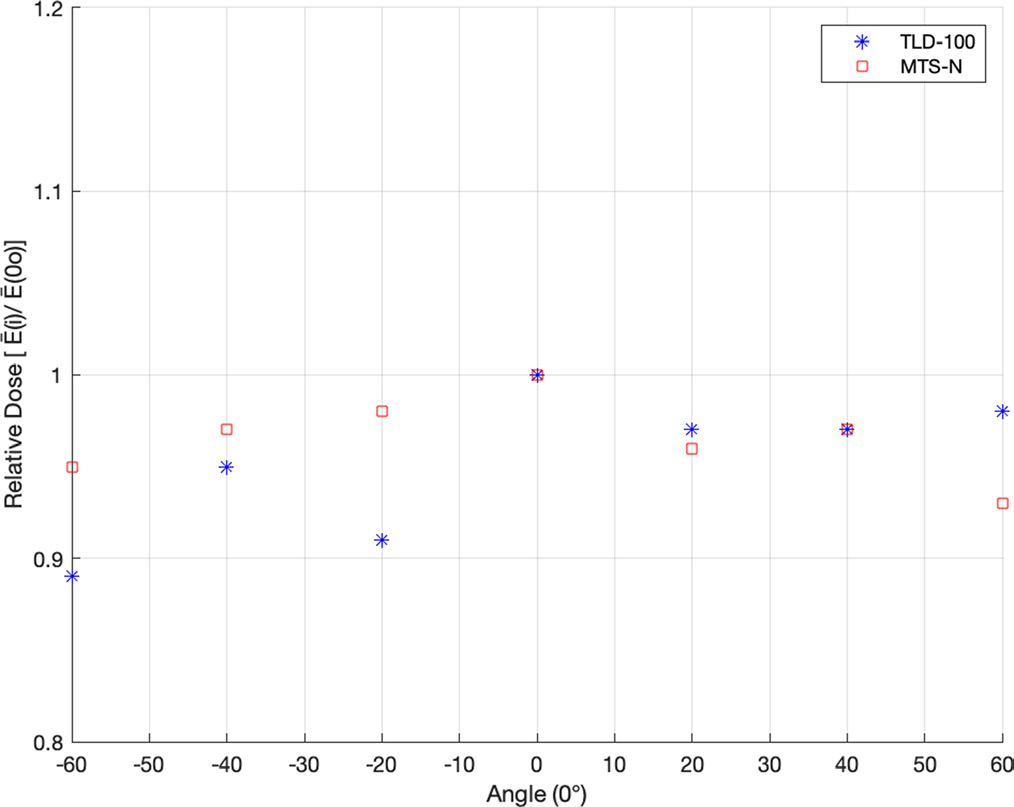
The dose response of both TLDs types due to variation of angles relative to 0° incident angle using Cs-137 radiation with a dose of 10 mSv.

To cover a wide angular range, the relative dose was evaluated for Cs-137 radiation with a dose of 10 mSv by varying the angle of incidence in relative to the normal, from 60°, 40°, 20°, 0°, −20°, −40°, −60°. From bottom of Figure 5, MTC-N had a relative dose of about 0.89 with a maximum deviation from the response of the normal 11.26% at the angle of −60, whereas TLD-100 had a relative dose of about 0.93 with a maximum deviation from the response of the normal 6.68% at the angle of 60, and both did not exceed the maximum acceptable limit of 15% according to the IEC 61066 International Standard.^11^ The MTS-N shows better angular dependency than the TLD-100 within 4.58%.

### Effect of temperature

A total of 30 TLDs were irradiated at dosages of 1, 2, and 3 mSv and were kept at a nearly-room-temperature of 25°C as the initial control group for 24 h and subsequently were read. This procedure using the same TLDs in previous test was repeated after storing them in 45°C and 65°C in an oven separately. High temperature surroundings show a significant vulnerability to signal loss, according to the dose response results from the two TLDs, TLD-100 and MTS-N. This can be shown in Table 8, which shows that when the post-irradiation temperature increases, the TLD signal decreases. The average signal loss for the TLD-100 is 7.4 and 4.3% for the MTS-N when the temperature difference is 20°C. The MTS-N outperforms the TLD-100 within nearly 12% less at the average of all doses at 65°C.

**Table 8.** Percentage differences of average TLD-100 and MTS-N signal fading based on temperature

Temperature	TLD-100			MTS-N		
	1 mSv	2 mSv	3 mSv	1m Sv	2 mSv	3 mSv
control	0%	0%	0%	0%	0%	0%
45°C	−10%	−8%	−18%	−2%	−1%	−12%
65°C	−20%	−20%	−28%	−6%	−7%	−15%

The above tests of the personal dosemeter TLDs have met the IEC 61066 criterion and were satisfactory compared to previous studies. The linearity values of the MTS-N is slightly higher than the TLD-100 values, both materials show linear behavior as indicated by the quality of the fit. The results in the Cs-137 beam are better as they are closer to unity. The intercept for the MTS-N is almost half the value of the intercept for TLD-100, this is consistent with the results that were found by Freire et al. (2008).^22^ The detector reproducibility represents the ability of each detector to maintain stable readings after successive use where the batch homogeneity of the TLD detectors represents the variation of each individual detector reading in relation to the mean reading value of the whole batch. The MTS-N gave more coefficients of variation than TLD-100 which indicate that TLD-100 provides higher stability than MTS-N for each detector separately. The reproducibility for all detectors combined all the used dosimeters showed satisfactory results in accordance to the performance test standards. The detector reproducibility was found below the maximum acceptable limit of 7.5% where the batch homogeneity showed a value below of 30%, these results agree with those found by Squair et al. (2007)^19^ where they showed that it is not to be considered a source of uncertainty if sensitivity correction factor of each detector is used. TLD-100’s light sensitivity results remained within the limits of not exceeding 0.1 mSv, however MTS-N did not, which is consistent with the reproducibility results, which show that MTS-N TLD has more variations than TLD-100. The angular dependence results for both detectors show that all dose responses are within the limits, it did not excess the acceptable value according to the IEC 61066 Standard which agrees with Luo et al. (2007) ^23^.Temperature effects on TLDs, on the other hand, are incompletely understood, as is their susceptibility to providing incorrect readings after being accidentally exposed to high temperatures. The experimental results of this study show that when the TLD-100 and MTS-N are exposed to temperatures of 45°C and 65°C, the reported doses extracted from the TLD-100 and MTS-N signals were reduced but both were below 30%.

## Conclusion

In this work, the dosimetric properties of two TLD detectors (TLD-100 and MTS-N) were evaluated at different energies and incidence angles. The findings were compared to the IEC 61066 standard. Different parameters, including TLD sensitivity inhomogeneity, reproducibility, linearity, energy dependency, angle dependence, light sensitivity (zero point), and fading have been assessed. This evaluation allowed the manufacturer’s stated dosimetric performance of the TLDs (TLD-100 and MTS-N) to be compared against technical radiation protection requirements. The experiments were carried out simultaneously for both TLDs, so that the results could be directly compared. For all tests, both the TLD-100 and the MTS-N fulfil the IEC 1066 standard. Finally, according to the IEC 61066 standard, the overall result for dosimetric properties determined in terms of dose equivalents for all combinations of detectors is acceptable and both dosimeters were shown to be suitable for detecting workers' external exposure to ionizing radiation. A future direction of this work could be more details study in the signal fading due to temperature and investigating other detectors such as the optically stimulated luminescence OSL according to the IEC 61066 standard.
